# Acid- and Volume-Sensitive Chloride Currents in Human Chondrocytes

**DOI:** 10.3389/fcell.2020.583131

**Published:** 2020-11-13

**Authors:** Michael Kittl, Martina Winklmayr, Katharina Helm, Johannes Lettner, Martin Gaisberger, Markus Ritter, Martin Jakab

**Affiliations:** ^1^Institute of Physiology and Pathophysiology, Paracelsus Medical University, Salzburg, Austria; ^2^Ludwig Boltzmann Institute for Arthritis and Rehabilitation, Paracelsus Medical University, Salzburg, Austria; ^3^Gastein Research Institute, Paracelsus Medical University, Salzburg, Austria

**Keywords:** acidic, chloride, current, chondrocytes, osteoarthritis, pH, volume, RVD

## Abstract

Chondrocytes face extreme alterations of extracellular osmolarity and pH, which force them to appropriately regulate their cell volume (CV) and cellular pH. Perturbations of these mechanisms lead to chondrocyte death and ultimately to osteoarthritis (OA), the most common chronic joint diseases worldwide. OA hallmarks are altered cartilage hydration and severe fluid acidification. Impaired CV regulation and acidotoxicity contribute to disease progression and volume-sensitive anion channels are upregulated in OA. This study assessed the effect of hypotonicity and extracellular acidification on chondrocyte Cl^–^ conductances and CV regulation. Cl^–^ currents and membrane potentials were measured in human C28/I2 cells and primary human chondrocytes using the patch clamp technique. Intracellular pH was assessed by BCECF fluorescence, CV measurements were performed using the Coulter method, and cell viability/cell death by a resazurin assay. Hypotonic cell swelling caused activation of a volume-sensitive outwardly rectifying (VSOR) Cl^–^ current followed by a regulatory volume decrease (RVD), which was attenuated by the Cl^–^ channel blocker DCPIB. Extracellular, but not intracellular acidification to pH ≤ 5.0 elicited an acid-sensitive outwardly rectifying (ASOR) Cl^–^ conductance. Activation of either current depolarized the cell membrane potential. Under simultaneous hypotonic and acidic stimulation, VSOR and ASOR currents transiently coactivated, giving rise to a mixed current phenotype. Over time the VSOR current gradually vanished and the residual conductance showed a pure ASOR current phenotype. Extracellular acidification caused an isotonic CV gain and a complete suppression of RVD under hypotonic conditions. The results suggest that deactivation of the VSOR current under acidic conditions impairs CV regulation in chondrocytes, which is likely to compromise chondrocyte viability.

## Introduction

Osteoarthritis (OA) is one of the most common chronic joint diseases worldwide. Its prevalence is increasing constantly due to aging of the population (Vos et al., 2012; Palazzo et al., 2016). As yet there are no disease-modifying drugs available and medical care is mainly limited to pain reduction. In severe cases of OA, joint replacement is the last therapeutic possibility. OA is characterized by (1) elevated catabolic responses, (2) inflammation of the synovial compartments, (3) subchondral bone alterations, and (4) osteophyte formation, which affect the whole joint and cause disability by progressive cartilage destruction (Man and Mologhianu, 2014). In view of the disease burden and the lack of remedies, studies on the onset and progression of OA as well as potential new targets for therapy are required.

Articular cartilage consists of extracellular matrix (ECM) and embedded chondrocytes, which are the sole cellular residents of the tissue. The ECM is highly hydrated with a water content of approximately 70% and hypertonic. Upon mechanical loadings, cartilage deformation, cell membrane stretch, and water extrusion lead to changes in both fluid volume and osmotic pressure (Maroudas et al., 1991; Sophia Fox et al., 2009). Under load, the osmotic concentration of the ECM can rise up to approximately 480 mosm/kg, which decreases again during relaxation (Lewis et al., 2011a). In this specific environment, chondrocytes need to actively adapt their cell volume (CV) based on the prevalent osmotic pressure. In general, a hypertonic challenge causes cell shrinkage, which is opposed by a gain of solutes and water through a process termed regulatory volume increase (RVI). Cells exposed to a hypotonic environment gain volume through passive water uptake and compensate cell swelling by extruding solutes, which osmotically drives the efflux of water [regulatory volume decrease (RVD)] (reviewed, e.g., in Lang et al., 1998; Hoffmann et al., 2009). By mediating the efflux of Cl^–^ and organic osmolytes, volume-sensitive outwardly rectifying (VSOR) anion channels/currents [also known as swelling-activated Cl^–^ currents, ICl_swell_, or volume-regulated anion currents (VRAC)] are critically involved in RVD in virtually any cell type. They contribute to the regulation of ion homeostasis, transepithelial transport, and electrical excitability, and are tightly linked to cell migration, phagocytosis, proliferation, and apoptosis (Jentsch et al., 2002; Jakab and Ritter, 2006; Okada et al., 2006; Hoffmann, 2011; Harl et al., 2013; Pedersen et al., 2016; Kittl et al., 2018). The function of the VSOR current in CV and membrane potential regulation has also been shown in chondrocytes (Wilkins et al., 2000; Isoya et al., 2009; Funabashi et al., 2010; Lewis et al., 2011a,b; Ponce et al., 2012; Kumagai et al., 2016), where the Cl^–^ current might indirectly affect matrix protein synthesis by modulating intracellular Ca^2+^ levels (Alford et al., 2003).

Importantly, the onset of OA is characterized by tissue swelling and enhanced hydration, leading to a decrease in osmolality and altered ionic composition (Bush and Hall, 2005). In OA patients, the osmolality of the synovial fluid is hypoosmotic (249–277 mosm/kg) compared to healthy subjects (295–340 mosm/kg) (Bertram and Krawetz, 2012). OA is also accompanied by an acidification of the extracellular space, which significantly affects cartilage metabolism and inhibits matrix synthesis (Wu et al., 2007; Sun et al., 2018). Under physiological conditions, the pH of cartilage is weakly acidic (pH 7.2–6.9), but under pathological conditions, the environment is further acidified by the production of pro-inflammatory factors and enhanced anaerobic glycolysis (Hall et al., 1996; Wilkins et al., 2000). Intraoperative *in situ* pH measurements revealed massive acidification in primary OA patients, ranging from pH 7.1 at stage 0 to pH 5.5 at stage 3 (Konttinen et al., 2002).

In different cell types, extracellular acidification elicits an acid-sensitive outwardly rectifying (ASOR) Cl^–^ current (Auzanneau et al., 2003; Nobles et al., 2004; Lambert and Oberwinkler, 2005; Yamamoto and Ehara, 2006; Wang et al., 2007; Kajiya et al., 2009; Kucherenko et al., 2009; Sato-Numata et al., 2013, 2014, 2016, 2017; Capurro et al., 2015; Kurita et al., 2015; Valinsky et al., 2017; Kittl et al., 2019). The ASOR current shows similarities to VSOR currents like its pharmacological profile, ion permeability sequence, and outward rectification. It was therefore debated if the two conductances are manifestations of the same channel protein(s) activated either by cell swelling or acidification, or if different entities constitute the VSOR and ASOR channel pore (Nobles et al., 2004; Lambert and Oberwinkler, 2005; Capurro et al., 2015). Most studies focused on whole-cell Cl^–^ currents either under hypotonic or acidic conditions, so that currently little is known about the functional interrelation and possible coexistence of the two currents under simultaneous hypotonic and acidic conditions. In a previous paper, we could show in microglial cells that VSOR and ASOR currents can be simultaneously active for a short period until the ASOR current eventually becomes dominating and we found that RVD was abrogated under acidic conditions in these cells (Kittl et al., 2019).

Cell swelling has been suggested to be one of the first changes in OA (Bush and Hall, 2003, 2005). Impaired CV regulation under hypoosmotic and acidic conditions prevailing in OA could therefore have a detrimental effect on chondrocyte viability and function and disease progression. The aim of our study was to investigate Cl^–^ conductances and CV regulation in chondrocytes under these conditions. We report on the functional expression of ASOR channels in human chondrocytes and possible effects of the interrelation of ASOR and VSOR Cl^–^ currents on CV homeostasis.

## Materials and Methods

### Salts, Chemicals, Drugs

All salts and chemicals were *p.a.* grade. Nigericin and tamoxifen were purchased from Sigma–Aldrich-Merck (Darmstadt, Germany), DCPIB (4-[(2-butyl-6,7-dichloro-2-cyclopentyl-2,3-dihydro-1-oxo-1H-inden-5 yl)oxy]butanoic acid) from Tocris (Abingdon, United Kingdom), and BCECF-AM [2’,7’-bis-(2-carboxyethyl)-5-(and-6)-carboxyfluorescein, acetoxymethyl ester] from Merck-Calbiochem (Darmstadt, Germany). Stock solutions of nigericin (5 mg/ml) and DCPIB (100 mM) were prepared in ethanol. Tamoxifen and BCECF-AM were dissolved in dimethyl sulfoxide (DMSO) to give stock solutions of 40 and 100 mM, respectively. Stocks were stored in aliquots at 20°C until use.

### Cell Culture

Human immortalized C28/I2 cells (Goldring et al., 1994; Finger et al., 2003) were cultured in 25 cm^2^ flasks with DMEM/HAM’s F-12 medium (Biochrom, Berlin, Germany) supplemented with 5% fetal bovine serum (FBS Superior, Biochrom) and antibiotic-antimycotic solution (100 U/ml penicillin, 0.1 mg/ml streptomycin, 0.25 μg/ml amphotericin-B; Sigma–Aldrich). C28/I2 cells were kept at 37°C in a humified atmosphere of 5% CO_2_ (standard culture conditions). Subcultures were established once a week until passage 25. Primary chondrocytes were isolated from total human knee arthroplasty samples with informed consent and ethical approval by the Ethics Committee of Salzburg (415-E/1965/4-2015) as described in Winklmayr et al. (2019). In brief, cartilage samples were removed from subchondral bone and crashed into small pieces. After washing with phosphate-buffered saline (PBS), they were put on a shaker and digested overnight in DMEM/HAM’s F-12 supplemented with FBS and 2 mg/ml collagenase (Thermo Fisher) at 37°C. After 24 h, cells were (1) centrifuged, (2) washed twice with PBS, (3) resuspended in medium, and (4) seeded as required for the following experiments. Primary chondrocytes were used up to passage 2.

### Patch Clamp

Cells were seeded on 0.01% poly-D-lysine (PDL)-coated coverslips (Ø 12 mm) and cultured for at least 24 h in DMEM/HAM’s F-12 medium. Coverslips were transferred to a RC-25 recording chamber (Warner Instruments, Hamden, CT, United States) and mounted on a Nikon Eclipse TE2000-U inverted microscope. Experiments were performed at room temperature in the whole-cell perforated patch clamp mode using 130 μM amphotericin to the pipette solution unless otherwise stated. Recordings were started as soon as the serial resistance was below 30 MΩ for the perforated configuration and below 10 MΩ if the ruptured configuration was applied. Patch electrode resistances were 4–9 MΩ. After establishing the whole-cell configuration, cells were superfused with an extracellular solution and data were recorded using an EPC-10 amplifier controlled by PatchMaster software (HEKA, Lambrecht/Pfalz, Germany). Cell membrane potential (*V*_mem_) recordings were performed in the zero-current clamp mode. The intracellular (pipette) solution contained (in mM): K_2_SO_4_ 70, NaCl 10, KCl 10, MgCl_2_ 4, CaCl_2_ 2, 4-(2-hydroxyethyl)-1-piperazineethanesulfonic acid (HEPES) free acid (FA) 5, ethylene glycol-bis(β-aminoethyl ether)-N,N,N’,N’-tetraacetic acid (EGTA) 10 (249 mosm/kg, pH 7.2 adjusted with KOH). The extracellular solution contained (in mM): NaCl 140, KCl 5.6, CaCl_2_ 2.5, MgCl_2_ 1.5, HEPES FA 10, glucose 4.5, and mannitol 5 (300 mosm/kg, pH 4.5 adjusted with HCl, pH 7.4 adjusted with NaOH). Voltage clamp recordings of Cl^–^ currents under neutral and acidic conditions were performed under symmetrical intra- and extracellular Cl^–^ conditions. The extracellular solution consisted of (in mM): NaCl 100, CaCl_2_ 2.5, MgCl_2_ 2.5, HEPES FA 10, and mannitol 80 (300 mosm/kg, pH 7.2 adjusted with NaOH). Mannitol was omitted to obtain a hypotonic (220 mosm/kg) solution for VSOR activation. To assess pH dependencies, the extracellular solution was titrated with HCl to a pH ranging from 4.5 to 5.0. We used HEPES FA buffer also for solutions with a pH < 5.0 since we did not find any difference to results obtained in control experiments using 2-(N-morpholino)ethanesulfonic acid (MES) hydrate. The pipette solution contained (in mM): CsCl 100, MgCl_2_ 5, HEPES FA 10, EGTA 11, raffinose 60, Mg-ATP 2 (303 mosm/kg, pH 7.2 adjusted with CsOH). The pipette solution was titrated to pH 4.5 (HCl) to assess the effect of an intracellular acidification. The currents were monitored in response to voltage ramps (500 ms duration, 10-s interval) and voltage steps (500 ms duration, increments of 20 mV) from −100 to +100 mV. The holding potential between the ramps/steps was 0 mV to desensitize voltage-activated currents. Bath solution exchange was performed with a valve-controlled gravity-driven perfusion system (ALA Scientific Instruments, Farmingdale, NY, United States) at a flow rate of 3–5 ml/min. Osmolalities of intra- and extracellular solutions were measured using a vapor pressure osmometer (Wescor, Logan, UT, United States).

### Cell Volume Measurements

C28/I2 cells were harvested by Trypsin/EDTA after growing under standard conditions. The cell suspension was split into aliquots, which were centrifuged for 4 min at 200 × *g*. The supernatants were discarded. Immediately before the first measurement (timepoint 0), the cell pellet was re-suspended in 20 ml of an extracellular solution containing (in mM): NaCl 100, KCl 5.6, CaCl_2_ 2.5, MgCl_2_ 1.5, HEPES FA 10, glucose 4.5, and mannitol 80 (300 mosm/kg). A hypotonic extracellular solution (220 mosm/kg) was obtained by the omission of mannitol. To measure the effect of acidification, the extracellular solution was adjusted to pH 7.4 and 4.5 titrated with NaOH or HCl, respectively. For the pH 4.5 solutions, MES hydrate was used instead of HEPES FA. DCPIB (10 μM) was added to the samples as indicated. The mean CVs (MCV in fl) in the different samples were alternately measured every 5 min over 60 min on a Beckman Coulter Z2 particle counter (Beckman Coulter, Krefeld, Germany) based on measuring changes in electrical resistance produced by non-conductive particles suspended in an electrolyte solution (Coulter method). Calibration for particle size was done using 10-μm Flow-Check fluorospheres (Beckman–Coulter). Data were analyzed with the Multisizer Software (Beckman–Coulter) using a 600-fl cutoff to exclude apoptotic cells and cell debris.

### Intracellular pH (pH_i_) Measurements

Cells were seeded in 96-well black microplates with clear bottom at a density of 1.5 × 10^5^ cells/ml and kept under standard conditions for 24–48 h. Cells were loaded with 5 μM of the membrane permeable pH-sensitive dye BCECF-AM for 30 min at 37°C in serum-free medium. After removal of the loading medium 100 μl of extracellular solution containing (in mM): NaCl 140, KCl 5.6, CaCl_2_ 2.5, MgCl_2_ 1.5, HEPES FA 10, glucose 4.5, and mannitol 5 (300 mosm/kg) with the pH adjusted to 7.4, 6.0, 5.0, or 4.5 were added into the wells in quadruplicates. Fluorescence measurements (bottom readings) were performed at 37°C in a humidity cassette on a Spark 20M multimode plate reader (Tecan, Austria). BCECF was alternately excited at 440 and 490 nm using the built-in monochromator and emission was measured at 535 nm (20-nm bandwidth, 40 μs integration time) every 5 min over 75 min. Readings from wells containing non-BCECF-loaded cells were used for background subtraction. 490/440 nm ratios calculated from the corrected fluorescence intensity values were converted to absolute pH using the high K^+^/nigericin calibration method (cells on the same microplate were exposed to solutions titrated to pH 8.0, 7.0, 6.0, or 5.0 in the presence of 140 mM KCl and 10 μg/ml nigericin; for pH interpolation, the 490/440 nm fluorescence ratios obtained from these samples were fitted with a second-order polynomial function).

### Cell Viability Assay

Viability measurements were performed as previously described by Helm et al. (2017). Briefly, after incubation for 2 or 4 h in an extracellular solution containing (in mM): NaCl 140, KCl 5.6, CaCl_2_ 2.5, MgCl_2_ 1.5, HEPES FA 10, glucose 4.5, and mannitol 5 (300 mosm/kg) with the pH adjusted to 7.4 or 7.0–3.0 in 0.5-pH-steps, cell viability was measured in each half of the 96-well microplate using the resazurin assay (7-hydroxy-3H-phenoxazin-3-one-10-oxide sodium salt; Sigma–Aldrich). The culture supernatant was replaced by 100 μl DMEM containing 0.5 mM resazurin (stock solution 2.5 mM in PBS). After 1 h of incubation at 37°C, 90 μl of the supernatant was transferred to a new 96-well microplate and stored at −20°C until measurement. The fluorescence of the product (resorufin) was detected at λ_ex_ = 535 nm and λ_em_ = 595 nm using a Zenyth microplate reader (Intensity-top-measurement; Anthos, Salzburg, Austria). Mean viability values were corrected for blank values (without cells) and related to untreated controls (UTC, without apoptosis inducer). All treatments were measured in quadruplicate wells.

### Statistics

Data are expressed as means ± standard error of the means (SEM) of at least three independent biological replicates (*n* ≥ 3). In all experimental series, solvent control samples were included. Statistical tests applied are specified in the figure legends. Means were considered significantly different at *p*-values < 0.05 (^∗^*p* < 0.05). Data were analyzed and plotted using GraphPad Prism 8 (GraphPad Software, La Jolla, CA, United States) or Igor Pro 8 (WaveMetrics, Portland, OR, United States).

## Results

### Extracellular Acidification Induces an Acid-Sensitive Outwardly Rectifying (ASOR) Cl^–^ Current

**FIGURE 1:**
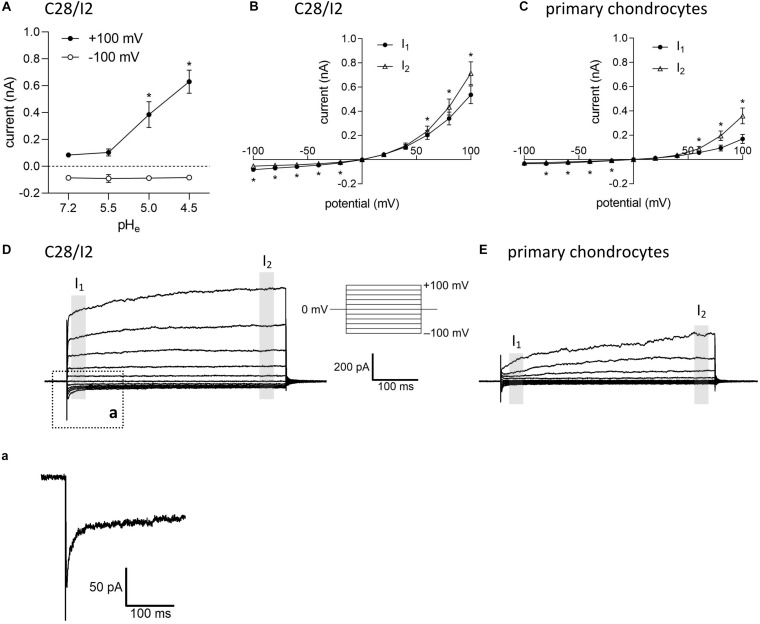
Extracellular acidification induces an acid-sensitive outwardly rectifying (ASOR) current in C28/I2 cells and primary human chondrocytes. **(A)** Mean amplitudes ± standard error of the means (SEM) of ASOR currents measured at pH 7.2 (*n* = 20), 5.5 (*n* = 4), 5.0 (*n* = 9), and 4.5 (*n* = 7). Asterisks indicate significant differences between pH 5.0 and pH 7.2 and between pH 4.5 and pH 7.2 (*p* < 0.05). Wilcoxon rank sum test. Current–voltage (*I*–*V*) relationships of mean ASOR currents ± SEM analyzed at the beginning (*I*_1_—open triangles) and at the end (*I*_2_—closed circles) of voltage pulses (indicated by gray shadings in **D** and **E**) in C28/I2 cells (*n* = 8) **(B)** and primary chondrocytes (*n* = 7) **(C)**. Asterisks indicate significant differences between *I*_1_ and *I*_2_ (*p* < 0.05). Paired *t*-tests. Examples of ASOR current traces elicited by 500-ms voltage steps from –100 to +100 mV in C28/I2 cells **(D)** and primary chondrocytes **(E)**. **(a)** Close-up of the inward current peak at –100 mV indicated in **(D)**.

Under control conditions (isotonic, pH 7.2), whole-cell currents in C28/I2 cells were small. Lowering the extracellular pH to ≤ 5.0 led to an increase in the whole-cell Cl^–^ conductance, which was fully reversible by reapplying control conditions. The current–voltage (*I*–*V*) relationship shifted from linear at pH 7.2 and 5.5 (baseline currents) to strongly outwardly rectifying at pH 5.0 and 4.5 (ASOR current). Figure 1A shows the mean current amplitudes recorded at pH 7.2, 5.5, 5.0, and 4.5. Using a voltage step protocol, currents were analyzed at the beginning and at the end of 500-ms voltage pulses (I_1_ and I_2_, respectively). In C28/I2 cells, the ASOR current amplitudes recorded at pH 4.5 showed time-dependent activation at constant positive holding potentials (+100 mV) and an initial negative current peak at −100 mV [Figures 1B,D and trace expansion (a)]. In primary chondrocytes, currents at pH 4.5 displayed the same characteristics; however, mean maximum current amplitudes were smaller as in C28/I2 cells (Figures 1C,E). Time-dependent changes in current amplitudes at constant holding potentials of +100 and −100 mV were quantified by calculating *I*_2_/*I*_1_ ratios with values > 1 indicating time-dependent activation and values < 1.0 indicating time-dependent inactivation as shown in Figures 3G, 4G for C28/I2 cells and primary chondrocytes, respectively. The time span for the ASOR current to reach peak values varied between the experiments, ranging from 5 up to 10 min (6.74 ± 0.65 min; *n* = 7).

### ASOR Current Activation Requires Extracellular but Not Intracellular Acidification

**FIGURE 2:**
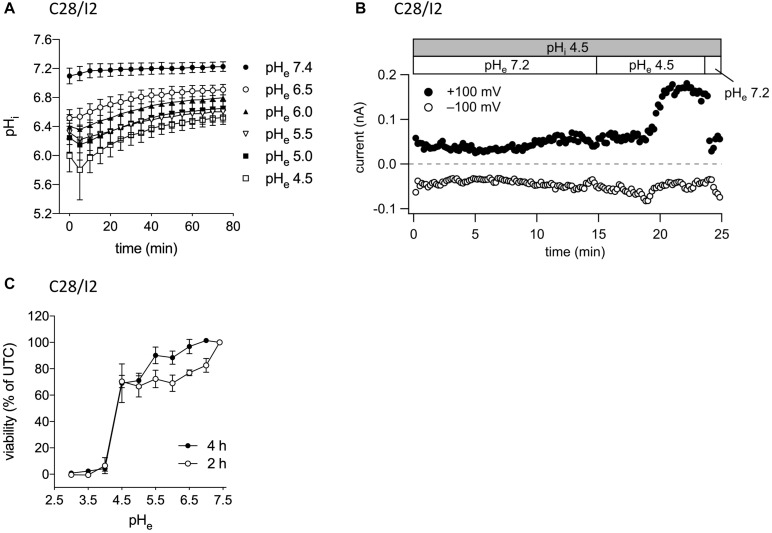
ASOR current activation requires extracellular but not intracellular acidification. **(A)** Intracellular pH measurements in C28/I2 cells at different timepoints after extracellular acidification (pH 6.5, 6.0, 5.5, 5.0, and 4.5) *versus* control conditions (pH 7.4); means ± SEM (*n* = 6). **(B)** ASOR current time course in C28/I2 cells (single experiment) using an acidic intracellular (pipette) solution (pH_i_ 4.5). Each circle represents the current at +100 mV (upper trace) and –100 mV (lower trace) measured in response to 500-ms voltage ramps applied every 10 s. The recording was performed in the ruptured patch clamp configuration. **(C)** Viability of C28/I2 cells after 2 and 4 h of exposure to pH 3.0, 3.5, 4.0, 4.5, 5.0, 5.5, 6.0, 6.5, 7.0, and 7.4; means ± SEM (*n* = 3–4).

In their native environment, chondrocytes are confronted with a substantial acidic load. In addition to intracellular acid loading due to lactate production under hypoxic conditions, there is constant leakage of H^+^ ions into the cells from the acidic extracellular space (Wilkins et al., 2000). To test, in how far exposure to a low extracellular pH (pH_e_) leads to an intracellular acidification in our experimental system, we monitored the intracellular pH (pH_i_) in C28/I2 cells by using the pH-sensitive dye BCECF. Immediately after reducing pH_e_ to 6.5, 6.0, 5.5, 5.0, or 4.5, the pH_i_ significantly decreased to pH 6.5–5.8, depending on the degree of extracellular acidification, but did not reach values as low as the prevailing pH_e_. After 5–10 min, the pH_i_ started to slowly but not fully recover within 80 min to values of 6.5–6.9 (Figure 2A). The decline in the pH_i_ might be sensed by ASOR channels and cause their activation. Therefore, we next investigated in C28/I2 cells, if intracellular acidification elicits the ASOR current. For this purpose, we performed patch clamp experiments in the ruptured configuration and used an acidic pipette solution (pH 4.5) to acidify the intracellular milieu. As shown in Figure 2B, decreasing the pH_i_ *via* the pipette solution neither affected basal whole-cell currents nor elicited an ASOR current unless an acidic extracellular solution was applied, which shows that only extracellular acidification can trigger ASOR current activation. Furthermore, cell viability measurements revealed that C28/I2 cells tolerate extracellular acidification to pH values as low as 4.5 for an extended period (4 h). Below a pH_e_ of 4.5 cell viability steeply declined and virtually all cells died (Figure 2C). We therefore did not expose cells to a pH_e_ lower than 4.5.

### Extracellular Hypotonicity Induces a Volume-Sensitive Outwardly Rectifying (VSOR) Cl^–^ Current, Which Is Affected by Low pH_e_

**FIGURE 3:**
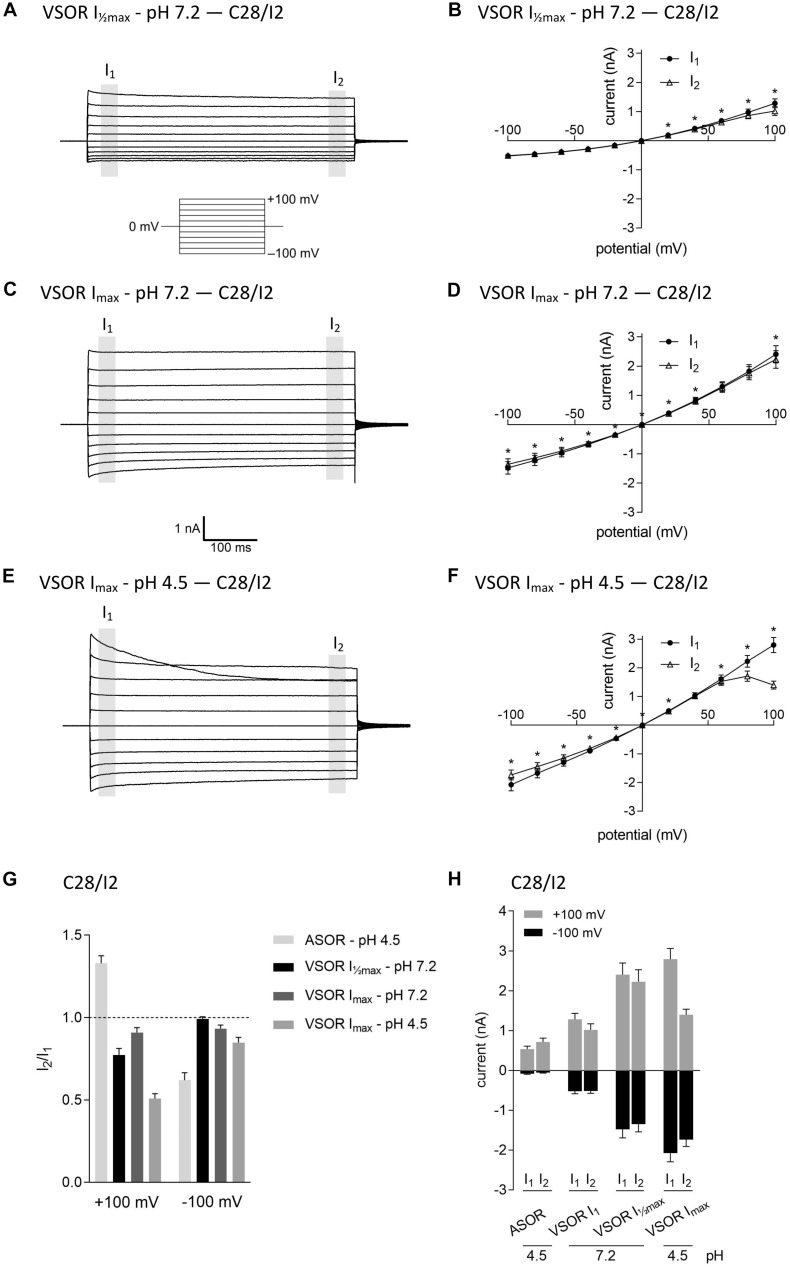
The volume-sensitive outwardly rectifying (VSOR) current in C28/I2 cells is affected by low pH. Examples of VSOR current traces elicited by 500-ms voltage steps from –100 to +100 mV obtained at half-maximal VSOR current amplitudes (VSOR *I*_max_—pH 7.2) **(A)**, at maximal VSOR current amplitudes (VSOR *I*_max_—pH 7.2) **(C)**, and at maximal VSOR current amplitudes immediately after reducing pH_e_ to 4.5 (VSOR *I*_max_—pH 4.5) **(E)**. **(B,D,F)** *I*–*V* relationships of mean VSOR currents analyzed at the beginning (*I*_1_—closed circles) and at the end (*I*_2_—open triangles) of voltage pulses (indicated by gray shadings in **A**, **C**, and **E**). **(B)** VSOR *I*_max_—pH 7.2 (*n* = 10). **(D)** VSOR *I*_max_—pH 7.2 (*n* = 11). **(F)** VSOR *I*_max_—pH 4.5 (*n* = 9). Asterisks indicate significant differences between *I*_1_ and *I*_2_ (*p* < 0.05). Paired *t*-tests. **(G,H)** *I*_2_/*I*_1_ ratios and mean current amplitudes, respectively, of ASOR currents and VSOR *I*_max_—pH 7.2, VSOR *I*_max_—pH 7.2, and VSOR *I*_max_—pH 4.5 currents at +100 and –100 mV. Means ± SEM (*n* = 9–11). *I*_2_/*I*_1_ ratio > 1, time-dependent activation; *I*_2_/*I*_1_ ratio < 1, time-dependent inactivation.

**FIGURE 4:**
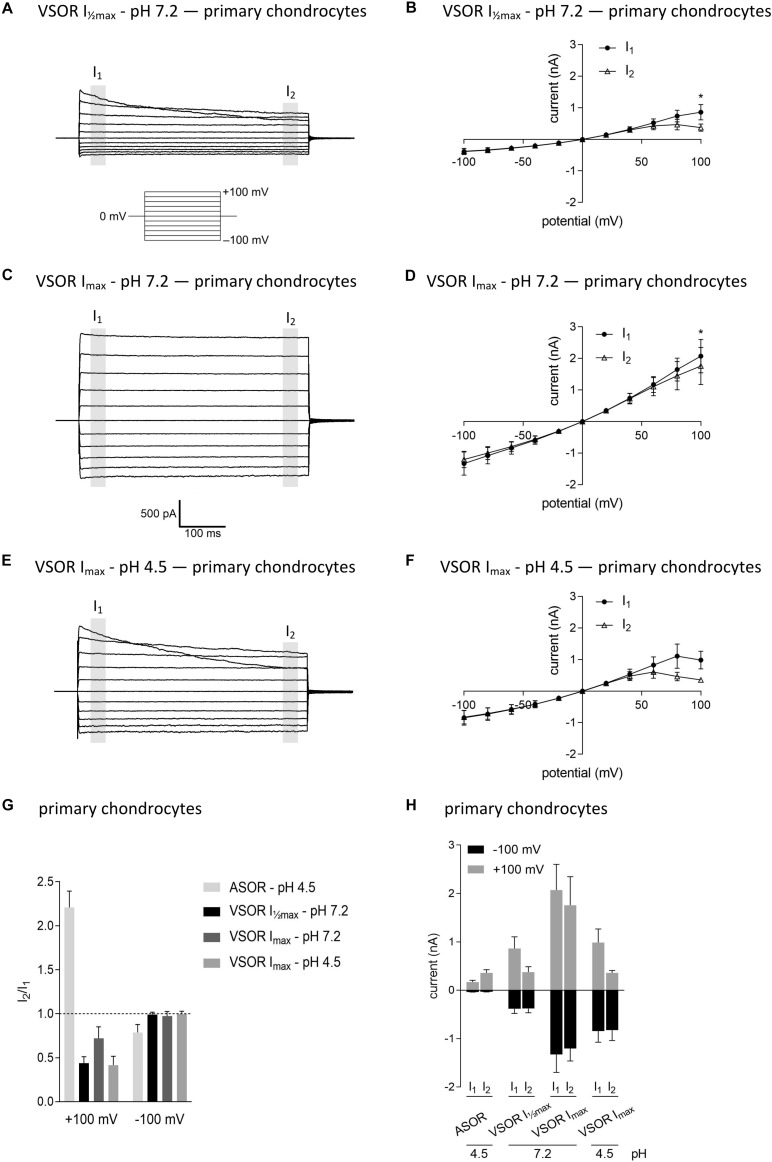
The volume-sensitive outwardly rectifying (VSOR) current in primary chondrocytes is affected by low pH. VSOR current traces elicited by 500-ms voltage steps from –100 mV to +100 mV obtained at half-maximal VSOR current amplitudes (VSOR *I*_max_—pH 7.2) **(A)**, at maximal VSOR current amplitudes (VSOR *I*_max_—pH 7.2) **(C)**, and at maximal VSOR current amplitudes immediately after reducing pH_e_ to 4.5 (VSOR *I*_max_—pH 4.5) **(E)**. **(B,D,F)** *I*–*V* relationships of mean VSOR currents analyzed at the beginning (*I*_1_—closed circles) and at the end (*I*_2_—open triangles) of voltage pulses (indicated by gray shadings in **A**, **C**, and **E**). **(B)** VSOR *I*_max_—pH 7.2 (*n* = 5). **(D)** VSOR *I*_max_—pH 7.2 (*n* = 6). **(F)** VSOR *I*_max_—pH 4.5 (*n* = 3). Asterisks indicate significant differences between *I*_1_ and *I*_2_ (*p* < 0.05). Paired *t*-tests. **(G,H)** *I*_2_/*I*_1_ ratios and mean current amplitudes, respectively, of ASOR currents and VSOR *I*_max_—pH 7.2, VSOR *I*_max_—pH 7.2, and VSOR *I*_max_—pH 4.5 currents at +100 and –100 mV. Means ± SEM. **(G)** *n* = 3–7 (*I*_2_/*I*_1_ ratio > 1, time-dependent activation; *I*_2_/*I*_1_ ratio < 1, time-dependent inactivation). **(H)** *n* = 2–3.

In virtually all cells, swelling leads to the activation of volume-regulated anion channels (VRAC) also known as VSOR anion channels, which give rise to swelling-dependent Cl^–^ currents (termed ICl_swell_ or ICl_vol_) with amply described, characteristic features (reviewed, e.g., in Pedersen et al., 2016). As shown in Figures 3, 4, exposure of C28/I2 cells and primary chondrocytes, respectively, to a hypotonic extracellular solution (25% reduction in extracellular osmolality under pH 7.2) caused a reversible VSOR current activation. Compared to the ASOR current, the VSOR current was only weakly outwardly rectifying with an almost linear *I*–*V* relation at full current activation and showed a slower activation time course. While the ASOR current rapidly reached peak amplitudes, the VSOR current developed more slowly over time, reaching an activation plateau after approximately 10 min (10.17 ± 3.11 min; *n* = 9). During this slow activation, the VSOR current showed two different time-dependent manifestations at constant positive potentials (+100 mV). At half-maximal current amplitudes (*I*_max_), the current in both C28/I2 cells (Figures 3A,B,G) and primary chondrocytes (Figures 4A,B,G) displayed time-dependent inactivation, whereas at maximal current amplitudes (*I*_max_) time-dependent inactivation was no longer evident (Figures 3C,D,G, 4C,D,G). At half-maximal activation, VSOR currents at −100 mV were equal at *I*_1_ and *I*_2_, while at *I*_max_ the current slightly inactivated over time. We next investigated the response of maximal VSOR currents (VSOR *I*_max_) to strong acidification (VSOR *I*_max_—pH 4.5). Immediately after reducing pH_e_, time-dependent inactivation at positive potentials was accentuated in C28/I2 cells (Figures 3E,F) as well as in primary chondrocyte (Figures 4E,F). This is also reflected in Figures 3H, 4H showing lower mean amplitudes at *I*_2_ than at *I*_1_. At negative potentials (−100 mV) acidification did not significantly change the current phenotype. Mean ASOR currents in C28/I2 cells and primary chondrocytes were smaller than VSOR currents and the *I*_2_/*I*_1_ ratios were >1 at +100 mV and <1 at −100 mV, indicating time-dependent activation and inactivation at constant positive and negative potentials, respectively (Figures 3G,H, 4G,H).

### ASOR and VSOR Current Sensitivities to the Cl^–^ Channel Blockers DCPIB and Tamoxifen

**FIGURE 5:**
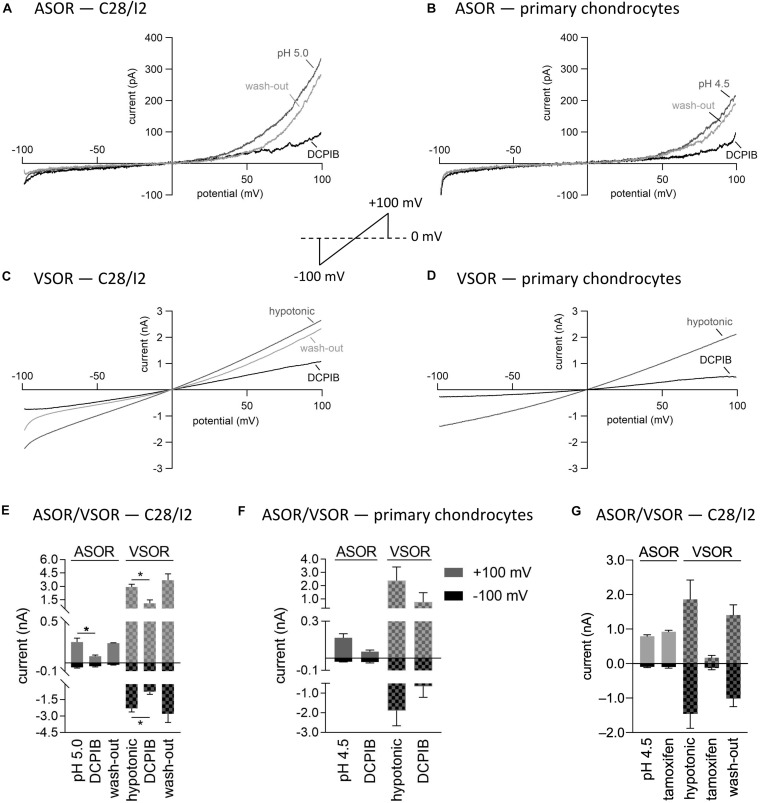
Volume- and acid-sensitive outwardly rectifying (VSOR and ASOR) current sensitivities to the Cl^–^ channel blockers DCPIB and tamoxifen. **(A,B)** and **(C,D)** Representative current–voltage relationship obtained by 500-ms voltage ramps under acidic and hypotonic conditions in the absence and presence of DCPIB (10 μM) in C28/I2 cells and primary human chondrocytes, respectively. **(E,F)** Mean peak currents ± SEM under acidic and hypotonic conditions in the absence and presence of 10 μM DCPIB in C28/I2 (*n* = 2–4) and primary chondrocytes (*n* = 2–3), respectively. **(G)** Mean peak currents ± SEM under acidic and hypotonic conditions in the absence and presence of 10 μM tamoxifen in C28/I2 (*n* = 3). Asterisks in **(E)** Indicate significant differences between pH 5.0 and DCPIB and between hypotonic and DCPIB (*p* < 0.05). Paired *t*-tests.

Next, we tested the Cl^–^ channel blocker DCPIB on the ASOR and VSOR current in C28/I2 cells and primary human chondrocytes. Data are shown in Figures 5A–F. While VSOR currents in wide range of cell types are efficiently blocked by DCPIB (for review see Pedersen et al., 2015), ASOR currents have been shown in two studies to be largely DCPIB-insensitive (Sato-Numata et al., 2016; Kittl et al., 2019). In the present study, ASOR and VSOR outward currents showed approximately the same sensitivities to 10 μM DCPIB at +100 mV, i.e., an inhibition of ∼65% in C28/I2 cells and ∼67% in primary chondrocytes. At −100 mV, the VSOR current was blocked by ∼68% in C28/I2 cells and ∼66% in primary chondrocytes, whereas the ASOR inward current was less sensitive with an inhibition of ∼18% in C28/I2 cells and no effect in primary chondrocytes. The block was reversible upon applying acidic or a hypotonic solution without DCPIB (not shown for primary chondrocytes). In accordance with previous work (Nobles et al., 2004; Yamamoto and Ehara, 2006; Sato-Numata et al., 2016), the VSOR channel blocker tamoxifen (10 μM) was ineffective on ASOR currents in C28/I2 cells, but reversibly suppressed both outward and inward VSOR currents by ∼90% (Figure 5G). Time courses of ASOR and VSOR current inhibition by DCPIB and tamoxifen are shown in Supplementary Figure S1.

### ASOR as Well as VSOR Current Activation Depolarizes the Cell Membrane Potential (*V*_mem_)

**FIGURE 6:**
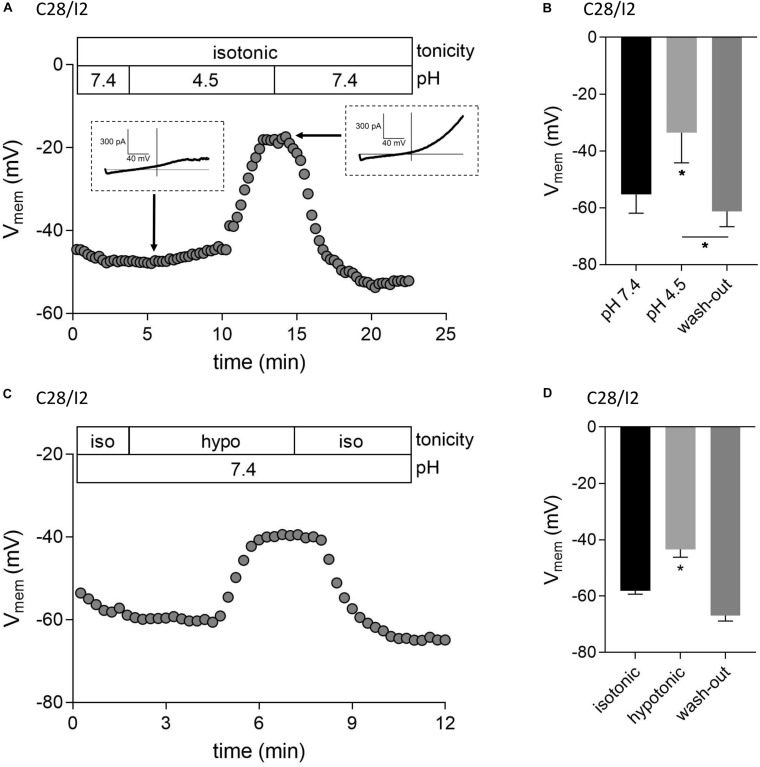
Acidic and hypotonic conditions lead to depolarization of the cell membrane potential (*V*_mem_). *V*_mem_ recordings of single C28/I2 chondrocytes at pH 7.4, pH 4.5, and pH 7.4 again (wash-out) **(A)**, and under isotonic (iso), hypotonic (hypo), and isotonic (wash-out) **(C)**. Each circle represents the mean *V*_mem_ obtained over 15 s. Insets in **(A)** show whole-cell Cl^–^ currents at the indicated timepoints in response to 500-ms voltage ramps from −100 to +100 mV from membrane potentials prevailing at the respective timepoints. **(B,D)** Mean *V*_mem_ ± SEM under the conditions given in **(A)** and **(C)** (*n* = 3 and *n* = 3–6, respectively). Asterisks in **(B)** indicate significant differences between pH 7.4 and pH 4.5 and between pH 4.5 and wash-out (*p* < 0.05). In **(D)**, the asterisk indicates a significant difference between isotonic and hypotonic conditions (*p* < 0.05). Paired *t*-tests.

We next tested if ASOR and VSOR current activation in C28/I2 cells affected the membrane potential (*V*_mem_). Figures 6A,C show examples of *V*_mem_ recordings before, during, and after the application of an acidic, or hypotonic solution, respectively. Mean *V*_mem_ values ± SEM under the respective conditions are shown in Figures 6B,D. The average resting *V*_mem_ under isotonic conditions and pH 7.4 was approximately −60 mV. Upon application of acidic (pH 4.5), or hypotonic (230 mosm/kg) extracellular solution, cells depolarized to ∼−34 mV (*n* = 3) and ∼−43 mV (*n* = 5), respectively. By switching from current- to voltage clamp during the acidity-induced depolarization, we verified ASOR current activation (insets in Figure 6A). The re-application of control solutions caused a rapid repolarization of *V*_mem_.

### Interplay Between ASOR and VSOR Currents—Effect of External Acidification on VSOR Currents

**FIGURE 7:**
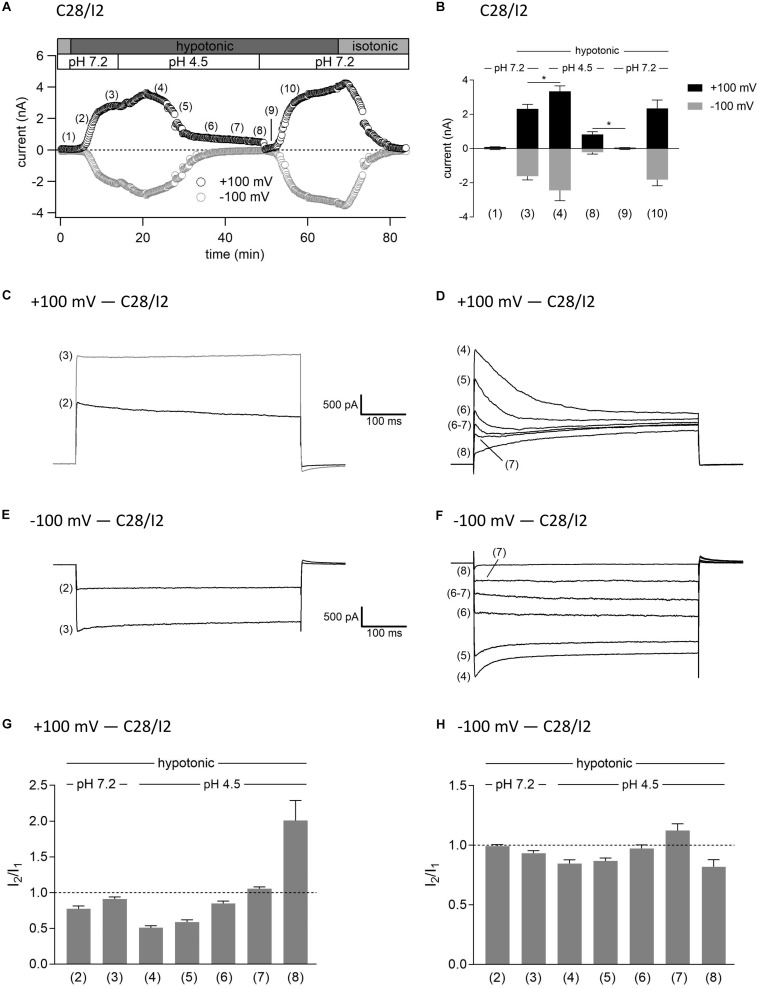
Interplay between ASOR and VSOR currents in C28/I2 cells—effect of external acidification on VSOR currents. **(A)** Time course of a single experiment under isotonic (300 mosm/kg) and hypotonic (220 mosm/kg) conditions at pH 7.2 or 4.5 as indicated. Each circle represents the current at +100 mV (upper trace) and –100 mV (lower trace) measured in response to 500-ms voltage ramps applied every 10 s. **(B)** Mean current amplitudes ± SEM measured at the timepoints (1)–(10) as indicated in **(A)** (*n* = 5–11). *p < 0.05 as indicated. Paired *t*-tests. Representative current traces in response to 500-ms voltage steps to +100 and –100 mV recorded at the timepoints marked in **(A)** at +100 mV **(C,D)** and –100 mV **(E,F)**. *I*_2_/*I*_1_ ratios ± SEM obtained at the timepoints indicated in **(A)** at +100 mV (*n* = 5–11) **(G)** and at –100 mV (*n* = 4–11) **(H)**; *I*_2_/*I*_1_ ratio > 1, time-dependent activation; *I*_2_/*I*_1_ ratio < 1, time-dependent inactivation.

**FIGURE 8:**
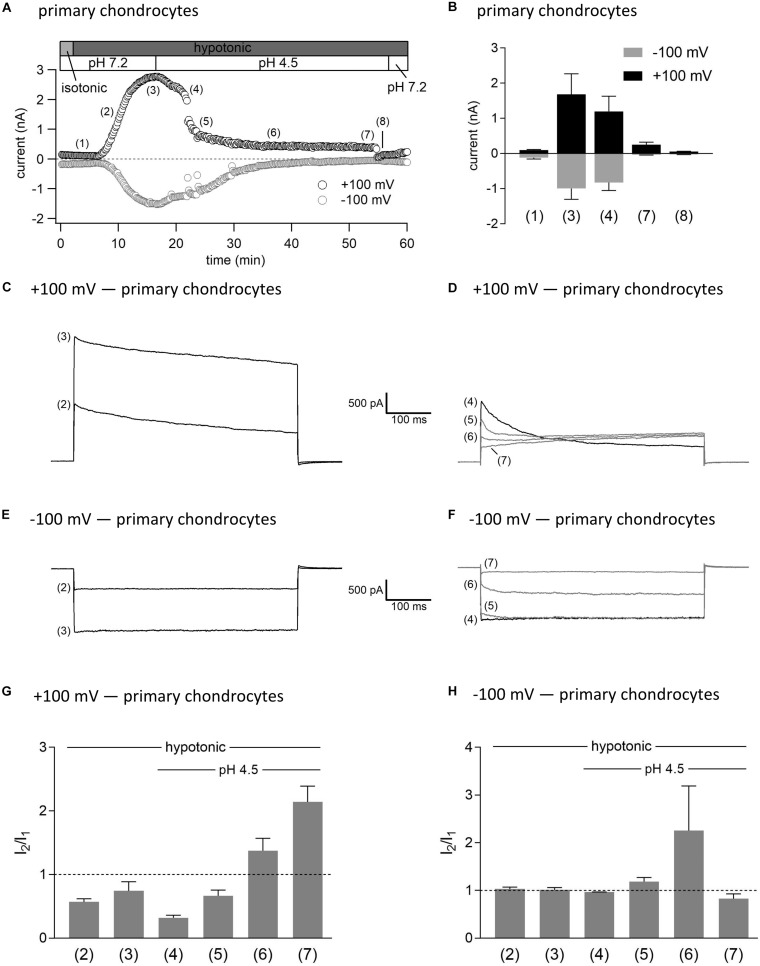
Interplay between ASOR and VSOR currents in primary human chondrocytes—effect of external acidification on VSOR currents. **(A)** Time course of a single experiment under isotonic (300 mosm/kg) and hypotonic (220 mosm/kg) conditions at pH 7.2 or 4.5 as indicated. Each circle represents the current at +100 mV (upper trace) and –100 mV (lower trace) measured in response to 500-ms voltage ramps applied every 10 s. **(B)** Mean current amplitudes ± SEM measured at the timepoints (1)–(8) as indicated in **(A)** (*n* = 3). Representative current traces in response to 500-ms voltage steps to +100 and –100 mV recorded at the timepoints marked in **(A)** at +100 mV **(C,D)** and –100 mV **(E,F)**. *I*_2_/*I*_1_ ratios ± SEM obtained at the timepoints indicated in **(A)** at +100 mV (*n* = 2–3) **(G)** and at –100 mV (*n* = 2–3) **(H)**; *I*_2_/*I*_1_ ratio > 1, time-dependent activation; *I*_2_/*I*_1_ ratio < 1, time-dependent inactivation.

As shown in Figures 3, 4, in both C28/I2 cells and primary chondrocytes, the acute effect of extracellular acidification to pH 4.5 on maximally activated VSOR currents was an acceleration of current inactivation at +100 mV. In a next set of experiments, we investigated the response of VSOR currents to prolonged extracellular acidification. Therefore, we first elicited the VSOR current by applying a hypotonic solution and then additionally lowered the external pH to 4.5 to test for a possible simultaneous, overlapping activation of both VSOR and ASOR currents. Results from C28/I2 cells and primary chondrocytes are shown in Figures 7, 8, respectively. Currents were measured every 10 s in response to voltage ramps and currents at +100 and −100 mV were plotted over time as shown in panels A. To analyze the prevailing current phenotypes, 500-ms current-steps from −100 to +100 mV were applied at 10 different timepoints (1)–(10) in C28/I2 cells and at eight different timepoints (1)–(8) in primary chondrocytes. Mean whole-cell Cl^–^ currents, original current tracings, and *I*_2_/*I*_1_ ratios at these timepoints are shown in panels B, C–F, and G–H, respectively.

Switching from isotonic [timepoint (1)] to hypotonic conditions at pH 7.2 was followed by a gradual VSOR current activation. At timepoint (2), when the VSOR current was half-maximally activated, a typical time-dependent inactivation at +100 mV was observed, which was absent at full current activation [timepoint (3)], similar as shown in Figures 3, 4 (*I*_2_/*I*_1_ ratio ∼1). At −100 mV, the VSOR current phenotype shifted during its activation from linear at half-maximal VSOR current amplitudes to a slight time-dependent inactivation over time at maximal VSOR current amplitudes. Additional acidification to pH 4.5 led to different responses in C28/I2 cells and primary chondrocytes. In C28/I2 cells, the whole cell Cl^–^ current increased, whereas in primary chondrocytes, a decrease was observed immediately after acidification [timepoint (4)]. The current phenotype at this timepoint, however, was similar in both cell types, displaying a steep inactivation at +100 mV and a moderate inactivation at −100 mV, similar as in Figures 3, 4 (panels E, F). Under continued acidity and hypotonicity, in both cell types, the whole-cell currents slowly decreased until a pure ASOR current appeared [C28/I2 cells: timepoint (8); primary chondrocytes: timepoint (7)], exhibiting typical time-depend current facilitation at +100 mV and an initial inward current peak at −100 mV. Current traces at timepoints (5), (6), and (7) in C28/I2 cells and timepoints (5) and (6) in primary chondrocytes showed mixed characteristics of ASOR and VSOR phenotypes with an initial inactivation followed by current activation over time at +100 mV and a slight time-dependent activation at −100 mV consistent with a progressive transition from an exclusive VSOR to a pure ASOR current. The *I*_2_/*I*_1_ ratio at +100 mV displayed a nadir at timepoint (4), followed by an increase during current superimposition [C28/I2 cells: timepoints (5), (6), and (7); primary chondrocytes: timepoints (5) and (6)] and a maximum, when the ASOR current became the dominating conductance (timepoints 8 and 7 in C28/I2 cells and primary chondrocytes, respectively). At −100 mV, the *I*_2_/*I*_1_ ratios show a U-shaped transition with two maxima at timepoint (2) and (7) and a minimum at timepoints (4) and (5) in C28/I2 cells, which was less pronounced in primary chondrocytes. In primary chondrocytes as well as in C28/I2 cells, the mean amplitude of the dominating ASOR current was significantly smaller than during the phase of current superimposition, indicating that the VSOR current was fully deactivated by strong acidification. This became evident after stepping back from pH 4.5 to pH 7.2 under continued hypotonic conditions, which caused a rapid ASOR current deactivation followed by a short timeframe at baseline current levels [C28/I2 cells: timepoint (9); primary chondrocytes: timepoint (8)]. Thereafter, the VSOR current slowly recovered from acidic inhibition to become fully reactivated [C28/I2 cells: timepoint (10); not shown for primary chondrocytes].

### Interplay Between ASOR and VSOR Currents—Effect of Hypotonicity on ASOR Currents

**FIGURE 9:**
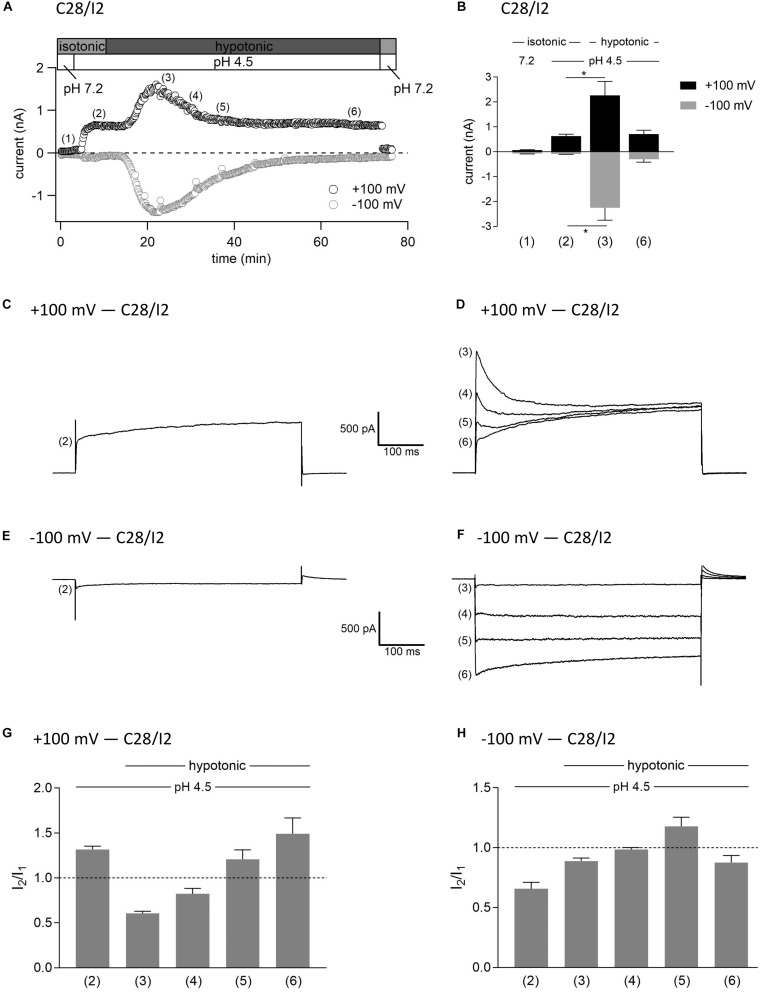
Interplay between ASOR and VSOR currents—effect of hypotonicity on ASOR currents in C28/I2 cells. **(A)** Time course of a single experiment under isotonic (300 mosm/kg) and hypotonic (220 mosm/kg) conditions at pH 7.2 or 4.5 as indicated. Each circle represents the current at +100 mV (upper trace) and –100 mV (lower trace) measured in response to 500-ms voltage ramps applied every 10 s. **(B)** Mean current amplitudes ± SEM measured at the timepoints (1)–(6) as indicated in **(A)** (*n* = 3–8). *p < 0.05 as indicated. Paired *t*-test. Representative current traces in response to 500-ms voltage steps to +100 and –100 mV recorded at the timepoints marked in **(A)** at +100 mV **(C,D)** and at –100 mV **(E,F)**. *I*_2_/*I*_1_ ratios ± SEM obtained at the timepoints as indicated in (A) at +100 mV (*n* = 5–11) **(G)** and at –100 mV (*n* = 4–11) **(H)**; *I*_2_/*I*_1_ ratio > 1, time-dependent activation; *I*_2_/*I*_1_ ratio < 1, time-dependent inactivation.

In this set of experiments on C28/I2 cells, we have chosen the opposite approach to test the effect of hypotonicity on the fully activated ASOR current. Figure 9A shows the time course of currents at +100 and −100 mV of a single experiment. Mean whole-cell Cl^–^ currents, original current tracings elicited by 500-ms voltage steps to +100 and −100 mV and *I*_2_/*I*_1_ ratios at timepoints (1)–(6) marked in panel A are shown in panels B, C–F, and G–H, respectively. By acidification we first activated the ASOR current to a stable plateau showing typical time-dependent activation over time at +100 mV [timepoint (2)] with an *I*_2_/*I*_1_ ratio > 1, and then simultaneously exposed the cells to hypotonicity. In a first phase, the whole-cell Cl^–^ current significantly increased further [timepoint (3)]. However, continued exposure to hypotonicity and acidity [timepoints (3)–(5)] caused a gradual decrease of the peak current to values measured under acidity alone [timepoint (6) compared to timepoint (2)]. Currents at maximum activation [timepoint (3)] showed steep inactivation over time at +100 mV, equal to the current phenotype of VSOR *I*_max_—pH 4.5 in Figure 3 and maximum currents in Figure 7. During the following declining phase, the whole-cell Cl^–^ current changed to a pure ASOR current phenotype with typical time-dependent facilitation at +100 mV [timepoint (6)]. Two transition states with characteristics of both the ASOR and VSOR current are shown at timepoints (4) and (5). This transformation is also evident from current tracings at −100 mV. The *I*_2_/*I*_1_ ratio at +100 mV gave values < 1 during superimposition of ASOR and VSOR current [timepoints (3) and (4)] and increased with progressive dominance of the ASOR current [values > 1 at timepoints (5) and (6)]. At −100 mV the *I*_2_/*I*_1_ ratio showed an inverted U-shaped transition with lowest values at timepoints (2) and (6) and a maximum at timepoint (5).

### Interplay Between ASOR and VSOR Currents—Effect of Simultaneous Exposure to Hypotonicity and Acidity

**FIGURE 10:**
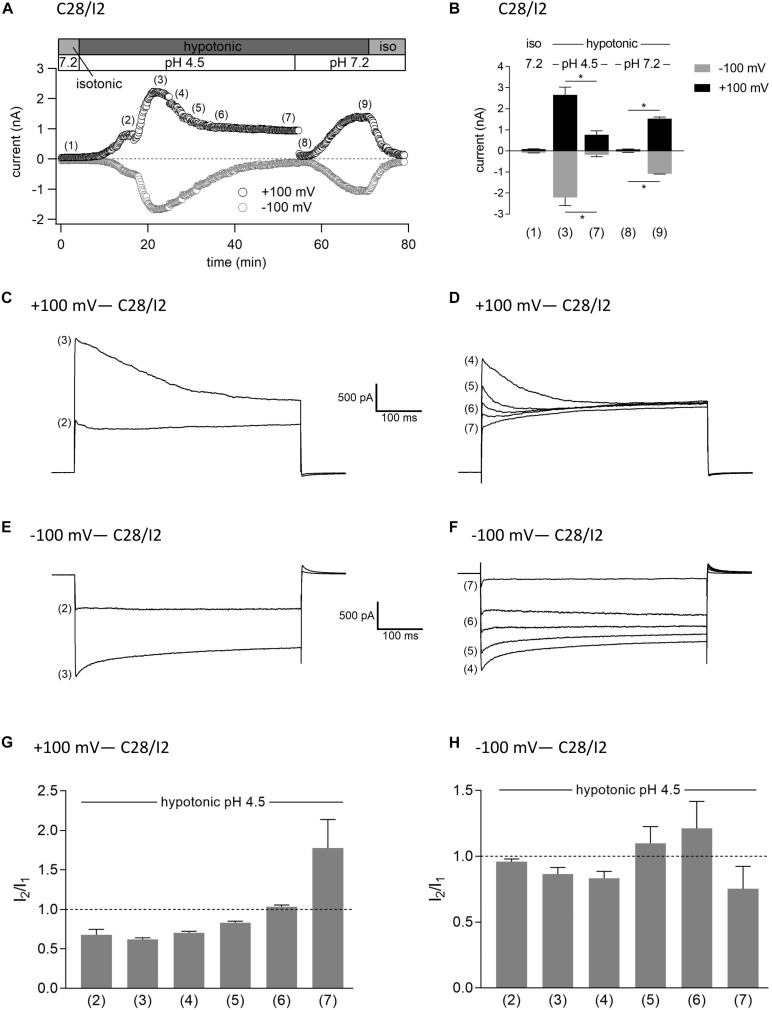
Interrelation between ASOR and VSOR currents—effect of simultaneous application of acidic and hypotonic conditions in C28/I2 cells. **(A)** Time course of a single experiment under isotonic (300 mosm/kg) and hypotonic (220 mosm/kg) conditions at pH 7.2 or 4.5 as indicated. Each circle represents the current at +100 mV (upper trace) and –100 mV (lower trace) measured in response to 500-ms voltage ramps applied every 10 s. **(B)** Mean current amplitudes ± SEM measured at the timepoints (1)–(9) as indicated in **(A)** (*n* = 3–6). *p < 0.05 as indicated. Paired *t*-tests. Representative current–voltage relationship obtained by 500-ms voltage ramps recorded at the timepoints marked in **(A)** at +100 mV **(C,D)** and at –100 mV **(E,F)**. *I*_2_/*I*_1_ ratios ± SEM obtained at the timepoints as indicated in **(A)** at +100 mV (*n* = 3–6) **(G)** and at –100 mV (*n* = 3–6) **(H)**; *I*_2_/*I*_1_ ratio > 1, time-dependent activation; *I*_2_/*I*_1_ ratio < 1, time-dependent inactivation.

In a last series of patch clamp experiments on C28/I2 cells, we investigated the activation kinetics of whole-cell Cl^–^ currents under simultaneous acidic and hypotonic conditions. Figure 10A shows the time course of currents at +100 and −100 mV of a single experiment. Mean whole-cell Cl^–^ currents, original current tracings at +100 and −100 mV and *I*_2_/*I*_1_ ratios at timepoints marked in panel A are shown in Figures 10B, C–F, and G–H, respectively. Current activation under hypotonic and acidic (pH 4.5) conditions resembled the activation time course of the VSOR current observed under hypotonic stimulation alone (Figure 7). However, time-dependent inactivation at +100 mV at maximum current activation [timepoint (3)] was more pronounced compared to hypotonic exposure alone (*I*_2_/*I*_1_ ratio < 1). This is most likely due to a superimposition of two processes—hypotonic VSOR current activation on the one hand and accelerated time-dependent current inactivation at positive potentials at low pH on the other hand as shown in Figure 3 (VSOR *I*_max_—pH 4.5) and at timepoint (4) in Figure 7D. Continued exposure to pH 4.5 and hypotonicity again led to a progressive decline in current amplitudes and a transformation to an exclusive ASOR current phenotype as observed in the previous experimental series shown in Figures 7–9, which is reflected in the current tracings and *I*_2_/*I*_1_ ratios at timepoints (4)–(7). After switching to normal extracellular pH (7.2), the ASOR current rapidly deactivated, and even though cells were still exposed to hypotonicity, whole-cell Cl^–^ currents transiently dropped to baseline levels [timepoint (8)] until the onset of VSOR current reactivation to a new maximum at timepoint (9).

### Effect of Acidification on Cell Volume Regulation

**FIGURE 11:**
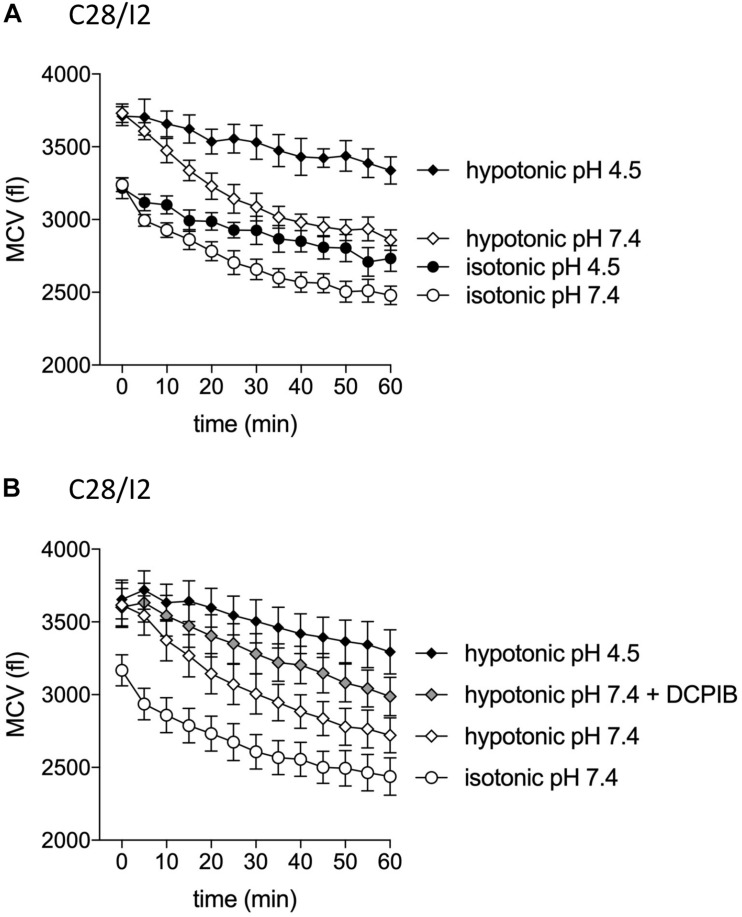
Cell volume regulation in C28/I2 cells is impaired under acidic conditions and in the presence of the Cl^–^ channel blocker DCPIB. **(A)** Mean cell volume (MCV) ± SEM measured over 1 h under isotonic (300 mosm/kg; circles) or hypotonic (220 mosm/kg; diamonds) conditions at pH 7.4 (open symbols) or pH 4.5 (black symbols) (*n* = 4). **(B)** MCV ± SEM measured over 1 h under in isotonic solution pH 7.4 (open circles), hypotonic solution pH 7.4 (open diamonds) in the absence and presence of DCPIB (10 μM; gray diamonds), and under hypotonic conditions at pH 4.5 (black diamonds) (*n* = 4). **(A)** and **(B)** show the results of two independent experimental series.

The VSOR current is essential for the RVD response by extruding Cl^–^ ions to counteract osmotic cell swelling. Given that the VSOR current gets deactivated by strong acidification while the ASOR current gets activated, we hypothesized that the cells’ volume regulatory ability might be affected at low pH. To test this, we performed CV measurements in C28/I2 cells under isotonic (300 mosm/kg) or hypotonic (220 mosm/kg) conditions at pH 7.4 or 4.5 and in the absence or presence of the Cl^–^ channel blocker DCPIB. Under isotonic pH 7.4 (control) conditions, we observed a 20–25% cell shrinkage from ∼3200 to ∼2450 fl (*n* = 4) (Figures 11A,B), which was attenuated under acidic (pH 4.5) conditions (shrinkage from ∼3200 to ∼2700 fl, i.e., a 15% volume loss; *n* = 4; Figure 11A). On average, at the first timepoint (0 min), the MCV of cells exposed to a hypotonic extracellular solution was ∼490 fl (Figure 11A) and ∼450 fl (Figure 11B) higher as under isotonic control conditions. After 60 min under hypotonic conditions and pH 7.4, the MCV was ∼380 and ∼280 fl higher compared to cells kept under isotonic conditions, respectively, indicating a moderate RVD response. Under hypotonic and acidic conditions (hypotonic pH 4.5), the difference in MCV after 1 h increased to ∼860 fl in both series of experiments. Similarly, RVD was impaired by DCPIB (10 μM); after 60 min the difference to the MCV measured under control conditions increased to ∼550 fl (Figure 11B).

## Discussion

In the present study we characterized an acid-sensitive and a volume-sensitive outwardly rectifying (ASOR and VSOR, respectively) Cl^–^ current in human C28/I2 chondrocytes and primary human chondrocytes. In detail, we investigated the mutual interdependence of the two currents and possible functional consequences of their interplay on chondrocyte CV regulation. Given their important role in CV homeostasis in chondrocytes on the one hand, and the relationship between deranged CV regulation, chondrocyte apoptosis, and OA on the other hand, Cl^–^ channels have come into focus of OA research (Roach et al., 2004; Isoya et al., 2009; Lewis et al., 2011a,b; Perez-Hernandez et al., 2012; Ponce et al., 2012; Kumagai et al., 2016; Yamamura et al., 2018; Hall, 2019).

Acid-sensitive outwardly rectifying currents with remarkably similar pH sensitivities, phenotypes, activation kinetics, and pharmacological profiles have been described in various cell types like microglia, myocytes, HeLa cells, neurons, Sertoli cells, distal tubular cells, erythrocytes, or osteoclasts (Auzanneau et al., 2003; Nobles et al., 2004; Lambert and Oberwinkler, 2005; Wang et al., 2007; Kajiya et al., 2009; Sato-Numata et al., 2013, 2014, 2016, 2017; Capurro et al., 2015; Valinsky et al., 2017; Kittl et al., 2019), but the knowledge on acid-sensitive Cl^–^ currents in chondrocytes is sparse. There is only one report on an ASOR-type Cl^–^ current in human OUMS-27 chondrocytes (Kurita et al., 2015). In line with these studies, we found the ASOR current in human C28/I2 and primary chondrocytes activated at an extracellular pH of ≤ 5.0, showed pronounced outward rectification and current facilitation (activation over time) at constant positive holding potentials. Intracellular acidification by dialysis of the cell with a pH 4.5-pipette solution did not activate the current, which is in accordance with previous studies on osteoclasts (Kajiya et al., 2009) and microglial cells (Kittl et al., 2019), and suggests an extracellular location of the proton binding site(s). Independence on intracellular pH is also supported by the finding that intracellular buffering of protons with 100 mM HEPES did not affect ASOR current activation in HEK293 cells (Lambert and Oberwinkler, 2005).

Volume-sensitive outwardly rectifying currents are activated by osmotic cell swelling and are ubiquitously existent in mammalian cells. Over the past decades, their biophysical characteristics, pharmacological profiles, and functions in CV regulation have been described in detail, as reviewed, e.g., in Jentsch et al. (2002), Jakab and Ritter (2006), Okada et al. (2006), Hoffmann (2011), and Pedersen et al. (2016). In the present study, we found that exposure to hypotonicity (230 mosm/kg) led to the activation of a typical VSOR Cl^–^ current in human articular chondrocytes, similar as previously described in rabbit and rat chondrocytes (Isoya et al., 2009; Ponce et al., 2012; Kumagai et al., 2016). Its inhibition by DCPIB impeded RVD during hypoosmotic challenge, which underlines the significance of the VSOR current for CV homeostasis in chondrocytes. Similar to the ASOR current, the VSOR current exhibited outward rectification, although less pronounced. The VSOR current showed typical time-dependent activation at constant positive holding potentials, which allowed a clear phenotypical discrimination from the ASOR current with its time-dependent activation at positive potentials. Inactivation of the maximally activated VSOR current (VSOR *I*_max_) over time at positive potentials became more pronounced immediately after lowering the extracellular pH, similar as previously observed in microglial cells (Kittl et al., 2019), which is also in line with earlier observations in *Xenopus* oocytes (Ackerman et al., 1994), C6 glioma cells (Jackson and Strange, 1995), BC3H1 mouse myoblasts (Voets et al., 1997), mouse neuroblastoma cells (Voets et al., 1997), or bovine pulmonary artery cells (Nilius et al., 1998). Increasing the extracellular concentration of Ca^2+^ and Mg^2+^ had a comparable effect on the inactivation kinetics (Voets et al., 1997), suggesting that Ca^2+^-, Mg^2+^-, and H^+^ ions might bind to the extracellular side of the channel to enhance time-dependent inactivation.

From numerous studies in many cell types, the ethacrynic acid derivative DCPIB is known as a potent VSOR current blocker (Pedersen et al., 2016). In our previous study on microglial cells (Kittl et al., 2019) and a study on HeLa cells by Sato-Numata et al. (2016), the VSOR current was virtually fully suppressed by 10 μM DCPIB, while at this concentration, the ASOR current was only inhibited by ∼20% in microglial cells and unaffected in HeLa cells. In the present study, however, we did not observe a difference in the DCPIB sensitivity between the VSOR and ASOR current. Both conductances were inhibited by 65–68% by 10 μM DCPIB in C28/I2 cells as well as in primary chondrocytes. In contrast to DCPIB, the sensitivities of ASOR and VSOR currents to tamoxifen were markedly different. In line with previous work on HeLa cells (Sato-Numata et al., 2016), HEK293 cells (Nobles et al., 2004), cardiac myocytes (Yamamoto and Ehara, 2006), and BV-2 microglial cells (Kittl et al., 2019), tamoxifen at 10 μM did not affect ASOR currents, but almost fully blocked VSOR currents by ∼90%.

Importantly, we found temporary ASOR and VSOR currents co-activation, when cells were simultaneously exposed to hypotonicity and acidic pH with superimposed currents and a mixed current phenotype. We observed this superimposition in three different experimental settings, and it was independent if (1) cells were first exposed to low pH to activate the ASOR current and then additionally to hypotonic conditions to activate the VSOR current, (2) cells were first exposed to hypotonic and then additionally to acidic conditions, or (3) cells were simultaneously exposed to hypotonic and acidic conditions. In each setting, after a period of current superimposition and under continued hypotonic conditions, we observed a transition to a pure ASOR current phenotype, indicating that the VSOR current was fully deactivated under low pH. The accelerated time-dependent inactivation of VSOR *I*_max_ at positive potentials observed immediately after exposure to pH 4.5 might be interpreted as the onset phase of VSOR current inactivation by low pH, which eventually leads to full current deactivation upon prolonged exposure to strongly acidic conditions.

Regarding the biophysical properties, activation kinetics, and DCPIB sensitivities of ASOR and VSOR currents, as well as the pH-sensitivity of the VSOR current and its complete deactivation under pH 4.5 we got identical results in the C28/I2 cell line—a well-established cell model that has been shown to phenotypically resemble articular chondrocytes (Goldring et al., 1994; Finger et al., 2003)—and in primary human chondrocytes. This suggests that our findings might show applicability also *in vivo*.

Including our previous work on microglial cells (Kittl et al., 2019), only few studies investigated the interrelation between ASOR and VSOR currents by combining hypoosmotic and acidic challenges. In line with our results in chondrocytes shown here, and our previous report on microglial cells (Kittl et al., 2019), Lambert and Oberwinkler (2005) provided evidence that ASOR and VSOR currents can be simultaneously active in HEK293T cells and reasoned that ASOR and VSOR channels are distinct populations of ion channels. In contrast, Nobles et al. (2004) postulated that ASOR and VSOR currents are manifestations of the same channel complex. Indeed, some biophysical similarities between ASOR and VSOR currents like outward rectification and ion permeability sequences might suggest a common molecular basis. However, there are distinct differences between ASOR and VSOR currents, such as more pronounced outward rectification of ASOR currents, current activation *versus* inactivation at constant positive membrane potentials, time courses of current activation, and differences in their pharmacological profiles. Studies in recent years, which identified members of the leucine-rich repeat containing 8 protein family (LRRC8 isoforms A-E; LRRC8A is also known as SWELL1) (Qiu et al., 2014; Voss et al., 2014; Sato-Numata et al., 2016, 2017; Syeda et al., 2016; Schober et al., 2017; Deneka et al., 2018) (reviewed in Pedersen et al., 2015; Stauber, 2015; Jentsch et al., 2016) and TMEM206 (Ullrich et al., 2019; Yang et al., 2019) as essential pore-forming components of VSOR and ASOR channels, respectively, provide compelling evidence that different molecular entities are underlying the two currents. Importantly, Sato-Numata et al. (2016, 2017) could show that LRRC8 family members are not involved in ASOR current activity. The reason for the acidity-induced deactivation and the functional and molecular interrelation of other proteins which might be associated with VSOR currents (Suzuki and Mizuno, 2004; Benedetto et al., 2016; Bae et al., 2019; Okada, 2019) needs further investigation.

Severe local acidosis is a hallmark of many diseases or disease-associated conditions including ischemia, cancer, and inflammation. In high-resolution measurements on tumor cell surfaces, Puppulin et al. (2018) could resolve highly localized variations of the proton concentration with an average pH of 6.7 and most acidic values of 5.1. OA is associated with chronic joint inflammation and acidification. Intraoperative *in situ* pH measurements in patients could show an OA stage-dependent cartilage acidification ranging from pH 7.1 at stage 0 to a value as low as pH 5.5 at stage 3 (Konttinen et al., 2002). Moreover, OA is associated with increased matrix hydration, reduced osmolality of the synovial fluid of 249–277 mosm/kg compared to 295–340 mosm/kg in healthy subjects (Bertram and Krawetz, 2012), and more pronounced hypoxia compared to physiological conditions (Milner et al., 2012). Chondrocytes reside in a bradytrophic, hypoxic, and acidic environment already under normal conditions, in which they constantly need to adapt their CV based on the prevalent osmotic pressures and mechanical loads (Hall et al., 1996; Wilkins et al., 2000). In OA, the developing hypoosmotic and acidic environment poses additional challenges to the chondrocytes’ CV and pH regulatory mechanisms. Therefore, we were interested in how far CV homeostasis in chondrocytes was affected under hypoosmotic or acidic conditions alone, and specifically in combination of both. Under isotonic conditions and normal pH, we observed a gradual cell shrinkage, which was attenuated under acidic conditions, similar as described in microglial cells (Kittl et al., 2019), HeLa cells (Wang et al., 2007), and cortical neurons (Sato-Numata et al., 2014). We assume that this was due to acid-induced counter-swelling caused by the activity of the Na^+^/H^+^ exchanger (NHE) (Behmanesh and Kempski, 2000) and probably by the activation of acid-sensing ion channels (ASICs) (Staunton et al., 2013) or other acid-sensitive cation channels expressed in chondrocytes, such as TRPM7 (Qian et al., 2019). Along with the ASOR current, their activation might also contribute to the depolarization of the membrane potential, which we could measure upon acidification.

In rabbit knee chondrocytes after anterior cruciate ligament transection (ACLT) as an OA model, Kumagai et al. (2016) found significantly higher VSOR current amplitudes, increased hypoosmotic cell swelling, and increased caspase 3/7 activity compared to sham surgery controls. These changes were evident prior to the onset of histologically apparent cartilage loss. In OA, the cartilage becomes hypocellular due to apoptosis and there is a clear correlation between the degree of cartilage damage and chondrocyte apoptosis (Hwang and Kim, 2015). Importantly, along with an increased number of primary lysosomes, autophagic vacuoles, and endoplasmic reticulum membranes, apoptosis in chondrocytes, termed chondroptosis, is associated with cell swelling rather than cell shrinkage [apoptotic volume decrease (AVD)] as a hallmark of classical apoptosis (Bush and Hall, 2003; Roach et al., 2004; Okada et al., 2006; Hwang and Kim, 2015; Hall, 2019).

In any case, chondrocyte swelling arising from reduced tissue osmolarity in OA is likely to augment the risk of cell damage or cell death during mechanical loading, since chondrocytes in swollen cartilage are highly sensitive to impact load (Hall, 2019). Tissue acidification during OA could adversely add to the deleterious effect of hypotonic stress, might lead to a breakdown of chondrocyte CV homeostasis, and push chondrocytes toward cell death. We found in two experimental series higher mean CVs when cells were concomitantly exposed to acidic and hypotonic conditions compared to sole hypoosmotic exposition. We assume that this resulted from acidotoxic cell swelling as observed under isotonic conditions plus an impaired RVD due to acidity-induced deactivation of VSOR channels. Therefore, we conclude that (1) VSOR current deactivation by acidity has similar functional consequences as the pharmacological inhibition of the current by DCPIB and (2) ASOR channels activated under acidic conditions cannot functionally replace VSOR channels in driving an RVD response.

Summarizing our findings, this study shows that CV homeostasis of chondrocytes is massively impaired under acidic and hypotonic conditions, which are characteristic for the osteoarthritic cartilage. Cell swelling under these conditions is likely to compromise cell viability and promote apoptosis. Since the maintenance of the ECM exclusively depends on chondrocytes, compromised CV homeostasis and viability are crucial factors promoting articular cartilage degeneration and the progression of OA.

## Data Availability Statement

The original contributions presented in the study are included in the article/Supplementary Material. Further inquiries can be directed to the corresponding author.

## Ethics Statement

Primary chondrocytes were isolated from total human knee arthroplasty samples with informed consent and ethical approval by the Ethics Committee of Salzburg (415-E/1965/4-2015).

## Author Contributions

MK, MR, and MJ contributed to conceptualization. MK, MW, and MJ contributed to formal analysis. MJ contributed to funding acquisition. MK, MW, KH, JL, MG, and MJ contributed to investigation. MR and MJ contributed to supervision. MK, MW, MG, and MJ contributed to validation. MK contributed to visualization. MK and MJ contributed to writing–original draft. MK, MW, MG, MR, and MJ contributed to writing–review and editing. All authors contributed to the article and approved the submitted version.

## Conflict of Interest

The authors declare that the research was conducted in the absence of any commercial or financial relationships that could be construed as a potential conflict of interest.
