# Supplementary figures and images for: Acid- and Volume-Sensitive Chloride Currents in Human Chondrocytes
Source: Front Cell Dev Biol. 2020 Nov 13;8:583131. doi: 10.3389/fcell.2020.583131 (PMC7691427; doi:10.3389/fcell.2020.583131)

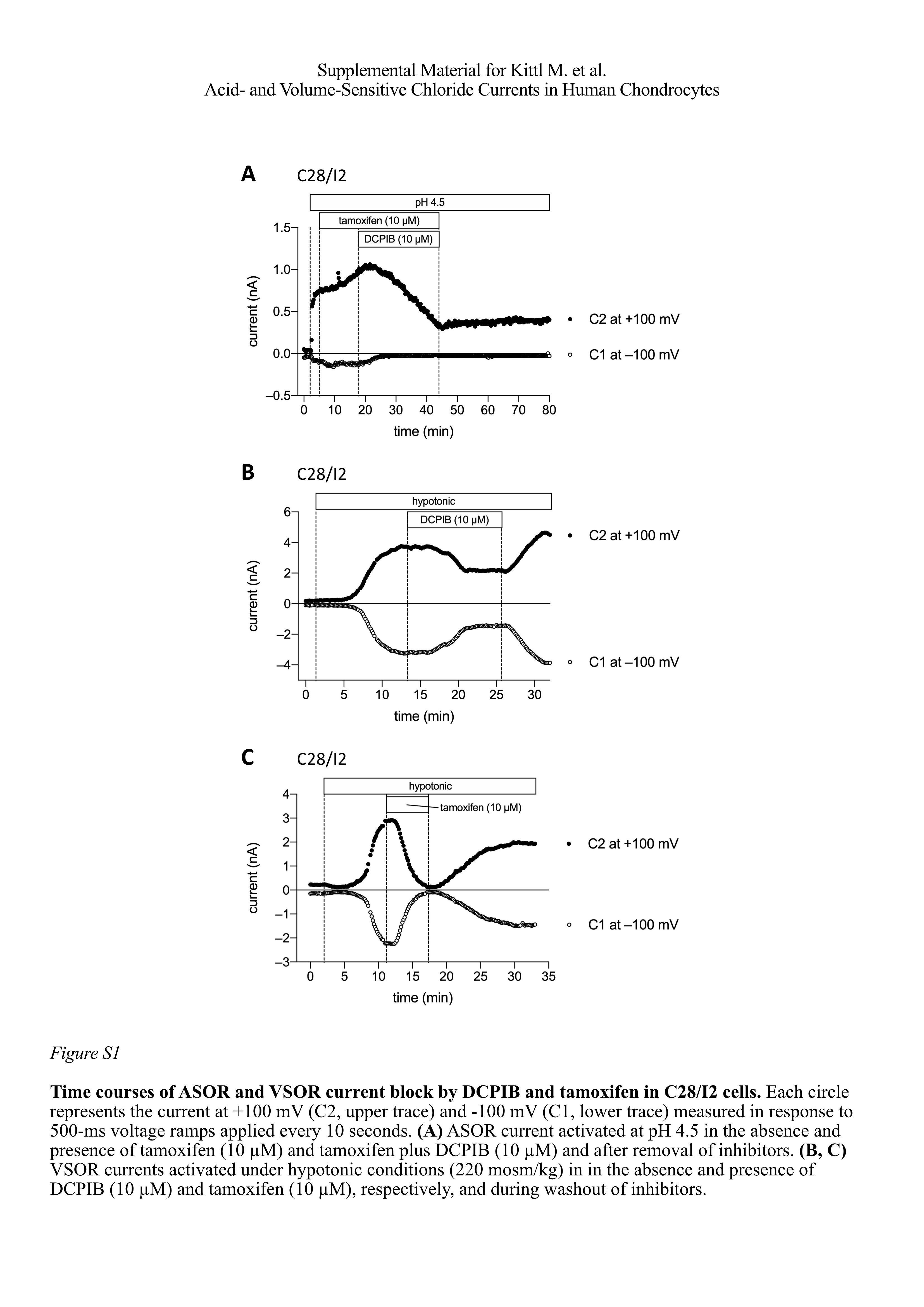

Supplement: Supplementary file 1 [file Image_1.JPEG]
